# Supplementary material for: Conserved arginine residues in synaptotagmin 1 regulate fusion pore expansion through membrane contact
Source: Nat Commun. 2021 Feb 3;12:761. doi: 10.1038/s41467-021-21090-x (PMC7859215; doi:10.1038/s41467-021-21090-x)
Supplement: Supplementary file 1 — Supplementary Information [file 41467_2021_21090_MOESM1_ESM.pdf]

Supplementary Information

Conserved Arginine Residues in Synaptotagmin 1 Regulate Fusion Pore Expansion Through Membrane Contact

Sarah B. Nyenhuis, Nakul Karandikar, Volker Kiessling, Alex J. B. Kreutzberger, Anusa Thapa, Binyong Liang, Lukas K. Tamm and David S. Cafiso

From the Departments of Chemistry, Molecular Physiology and Biological Physics, and the Center for Membrane Biology, University of Virginia, Charlottesville, VA.

Supplementary Table 1. Power Saturation parameters for spin labels in the arginine apex†

Label Position	lipid	metal added	depth parameter (Φ)	position from lipid phosphate (Å)	n
C2AB 285R1	Aqueous	none	–2.30, -2.31	aqueous	2
	POPC:POPS	Ca^{2+}	–1.62 ± 0.023	–2.2	3
		EGTA	–1.76 ± 0.05	–2.9	3
	POPC:PIP_2	Ca^{2+}	–2.04, -2.06	–4.7	2
		EGTA	–1.91, -1.95	–3.5	2
C2AB 349R1	Aqueous	none	–2.40, -2.41	aqueous	2
	POPC:POPS	Ca^{2+}	–1.73 ± 0.075	–2.0	6
		EGTA	–2.02 ± 0.064	–4.5	3
	POPC:PIP_2	Ca^{2+}	–2.16, -2.14	–5.6	2
		EGTA	–2.22, -2.27	–6.9	2
C2AB 350R1	Aqueous	none	–2.04, -1.98	aqueous	2
	POPC:POPS	Ca^{2+}	–1.76 ± 0.018	–2.2	3
		EGTA	–1.83 ± 0.034	–2.7	3
	POPC:PIP_2	Ca^{2+}	–1.61, -1.65	–1.1	2
		EGTA	–1.68, -1.60	–1.1	2

† Depth parameters and approximate label positions obtained by progressive power saturation of the EPR spectrum (see text, Methods). The number of independent samples and power saturation runs is given by n. Errors in the depth parameter are based upon standard deviations when n is ≥ 3. For cases where n=2, label positions are based upon the average of the two depth measurements.

Supplementary Figure 1

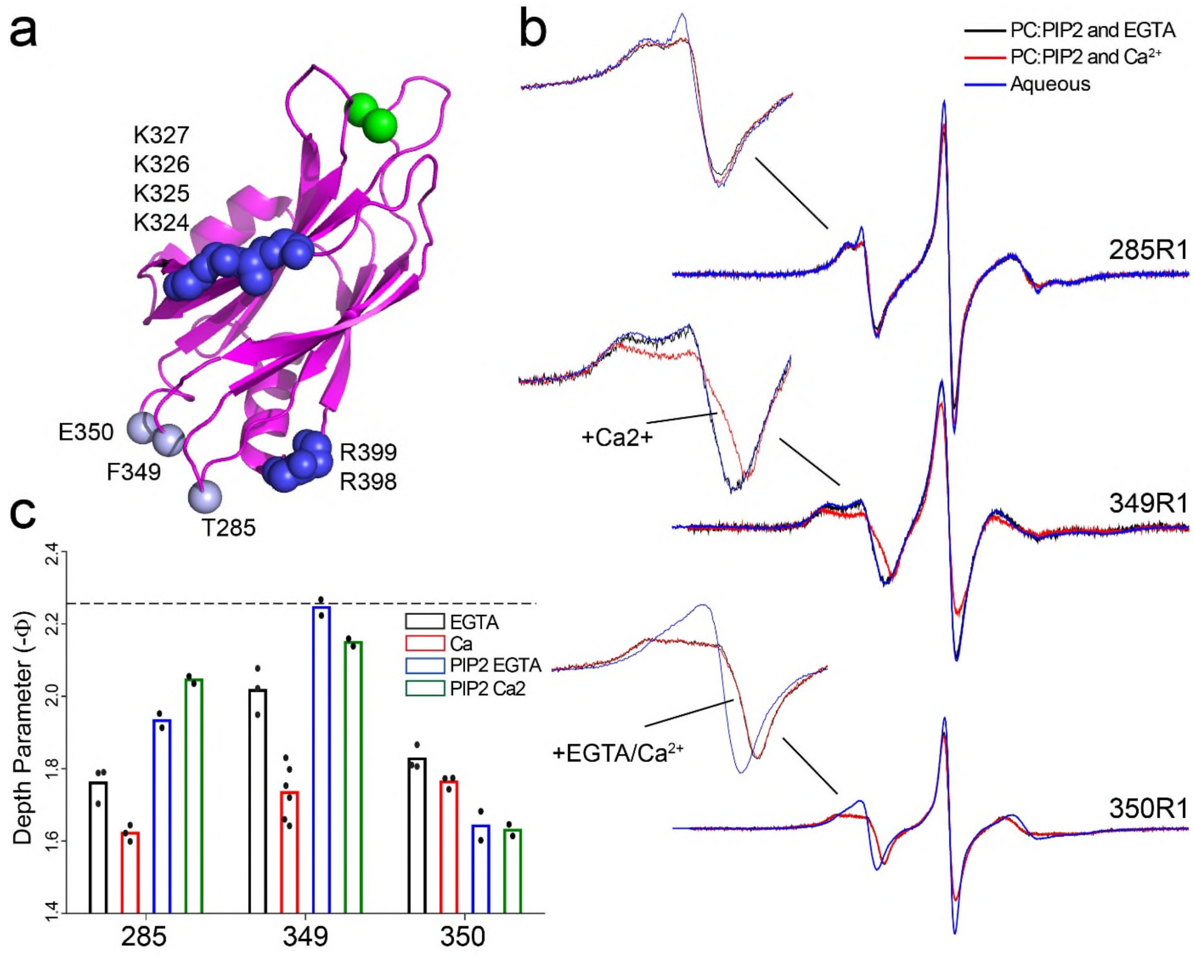

Supplementary Figure 1. **The arginine apex of C2B contacts the membrane interface when membranes contain PIP$_2$.** **a**) Model for the C2B domain of Syt1 showing the labeled residues near the arginine apex (R399, R398) as well as lysine residues in the polybasic face (K324-327). **b**) EPR spectra from sites near the apex without membranes (aqueous, blue traces), and in the presence of POPC:PIP$_2$ (95:5) bilayers with Ca^{2+} or EGTA (red and black traces, respectively). **c**) Membrane depth parameters comparing data for POPC:POPS (80:20) with data for POPC:PIP$_2$ (95:5), with EGTA or Ca^{2+}. The effect of Ca^{2+} is minimized in the presence of PIP$_2$, likely because the domain has a strong Ca^{2+}-independent association to PIP$_2$ containing membranes. Bar graphs indicate the mean values. Points represent power saturation runs on independent samples.

Supplementary Table 2. Power Saturation parameters for spin labels in the arginine apex with RQ and RQRQ mutations†

**Mutant/Label Position**	**lipid**	**metal added**	**depth parameter (Φ)**	**position from lipid phosphate (Å)**	**n**
C2AB 285R1 RQ	Aqueous	none	–2.29, -2.26	aqueous	2
	POPC:POPS	Ca^{2+}	–1.78 ± 0.028	–2.3	4
		EGTA	–1.96 ± 0.05	–2.9	4
	POPC:PIP_2	Ca^{2+}	–2.13, -2.18	–5.7	2
		EGTA	–2.04, -2.08	–4.7	2
C2AB 285R1 RQRQ	Aqueous	none	–2.26 ± 0.03	aqueous	2
	POPC:POPS	Ca^{2+}	–2.17 ± 0.008	–6.2	3
		EGTA	–2.19 ± 0.02	–6.4	3
	POPC:PIP_2	Ca^{2+}	–2.23, -2.27	aqueous	2
		EGTA	–2.25, -2.31	aqueous	2
C2AB 350R1 RQ	Aqueous	none	–2.04, -2.11	aqueous	2
	POPC:POPS	Ca^{2+}	–1.82, -1.85	–2.6	2
		EGTA	–1.92, -1.96	–3.5	2
	POPC:PIP_2	Ca^{2+}	–1.64, -1.67	–1.3	2
		EGTA	–1.66, -1.71	–1.5	2
C2AB 350R1 RQRQ	Aqueous	none	–2.08, -2.07	aqueous	2
	POPC:POPS	Ca^{2+}	–1.93, -1.97	–3.6	2
		EGTA	–2.00, -2.06	–4.3	2
	POPC:PIP_2	Ca^{2+}	–1.66, -1.71	–1.5	2
		EGTA	–1.68, -1.74	–1.6	2

† Depth parameters and approximate label positions obtained by progressive power saturation of the EPR spectrum (see text, Methods). The number of independent samples and power saturation runs is given by n. Errors in the depth parameter are based upon standard deviations when n is ≥ 3. For cases where n=2, the label position is based upon the mean of the two runs.

Supplementary Figure 2

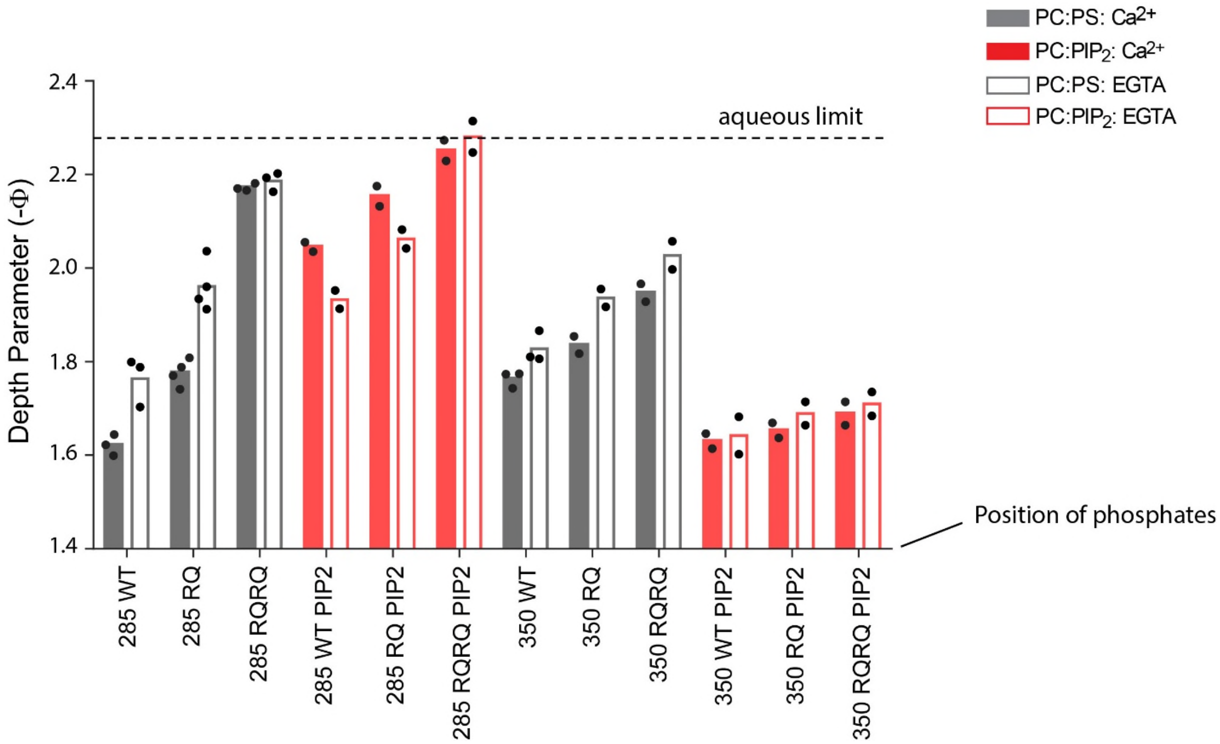

Supplementary Figure 2. Mutating the arginine apex reduces or eliminates membrane contact by the arginine apex in the absence of Ca^{2+}. A comparison of the membrane depth parameters for sites 285 and 350 for wild-type Syt1 and the RQRQ mutants in both the presence (data from Figure 3) and absence of Ca^{2+}. These power saturation data indicate that the RQ and RQRQ mutations reduce or eliminate membrane contact. Vesicles were composed of POPC:POPS (80:20) or POPC:PIP_2 (95:5). Bar graphs indicate the mean values. Points represent power saturation runs on independent samples.

Supplementary Figure 3

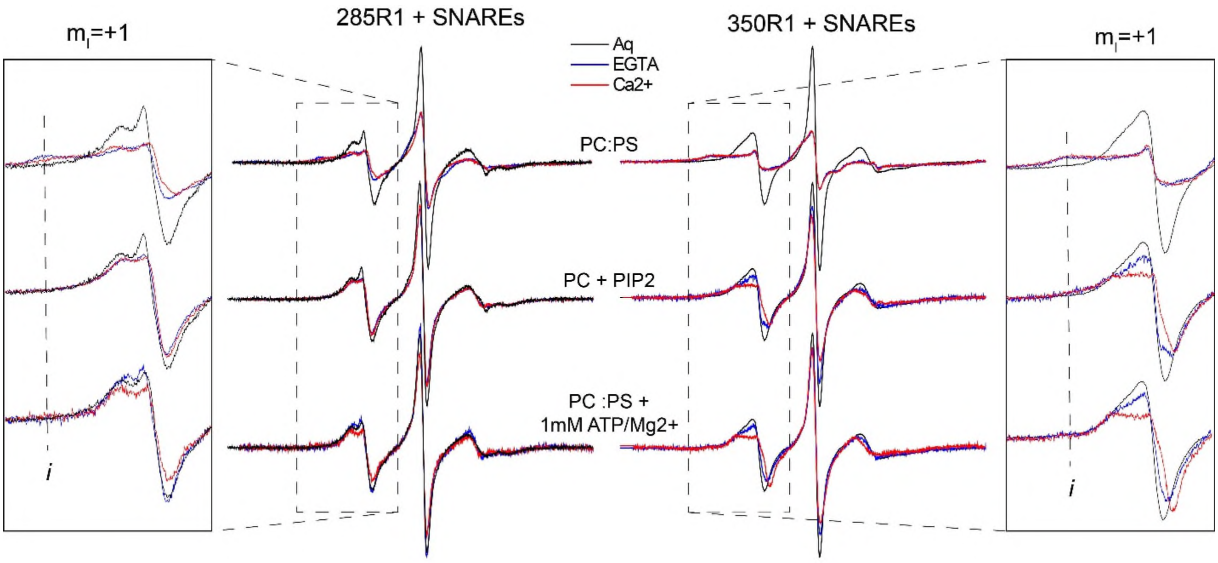

Supplementary Figure 3. *EPR spectra from 285R1 and 350R1 indicate that either ATP or PIP_2 eliminate contact with membrane reconstituted SNAREs*. In PC:PS, contact with the SNAREs is evident from the appearance of an immobilized component in the region of the m_I=-1 transition (see dashed line labeled "*i*") and the diminished normalized intensity of the EPR spectrum. However, when PIP_2 is present or when 1 mM ATP/Mg^{2+} is present, the interaction is no longer observed.

Supplementary Table 3. Power Saturation parameters for spin labels at the polybasic face of C2B†

Label Position	lipid conditions	metal added	depth parameter (Φ)	position from lipid phosphate (Å)
C2AB 323R1	Aqueous	none	-1.42, -1.49	aqueous
	POPC:POPS	Ca^{2+}	-0.804, -0.820	3.2
		EGTA	-1.38, -1.43	0.3
	POPC:PIP_2	Ca^{2+}	–1.25, -1.22	1.21
		EGTA	–1.28, -1.32	0.89
C2AB 329R1	Aqueous	none	–1.63, -1.68	aqueous
	POPC:POPS	Ca^{2+}	–1.28, -1.32	0.88
		EGTA	–1.36, -1.38	0.47
	POPC:PIP_2	Ca^{2+}	–1.21, -1.19	1.4
		EGTA	–0.298, -0.307	5.3

† Depth parameters and approximate label positions obtained by progressive power saturation of the EPR spectrum (see text, Methods). Two independent samples and power saturation runs were made for each condition. The label position is based on the average of two runs.

Supplementary Figure 4

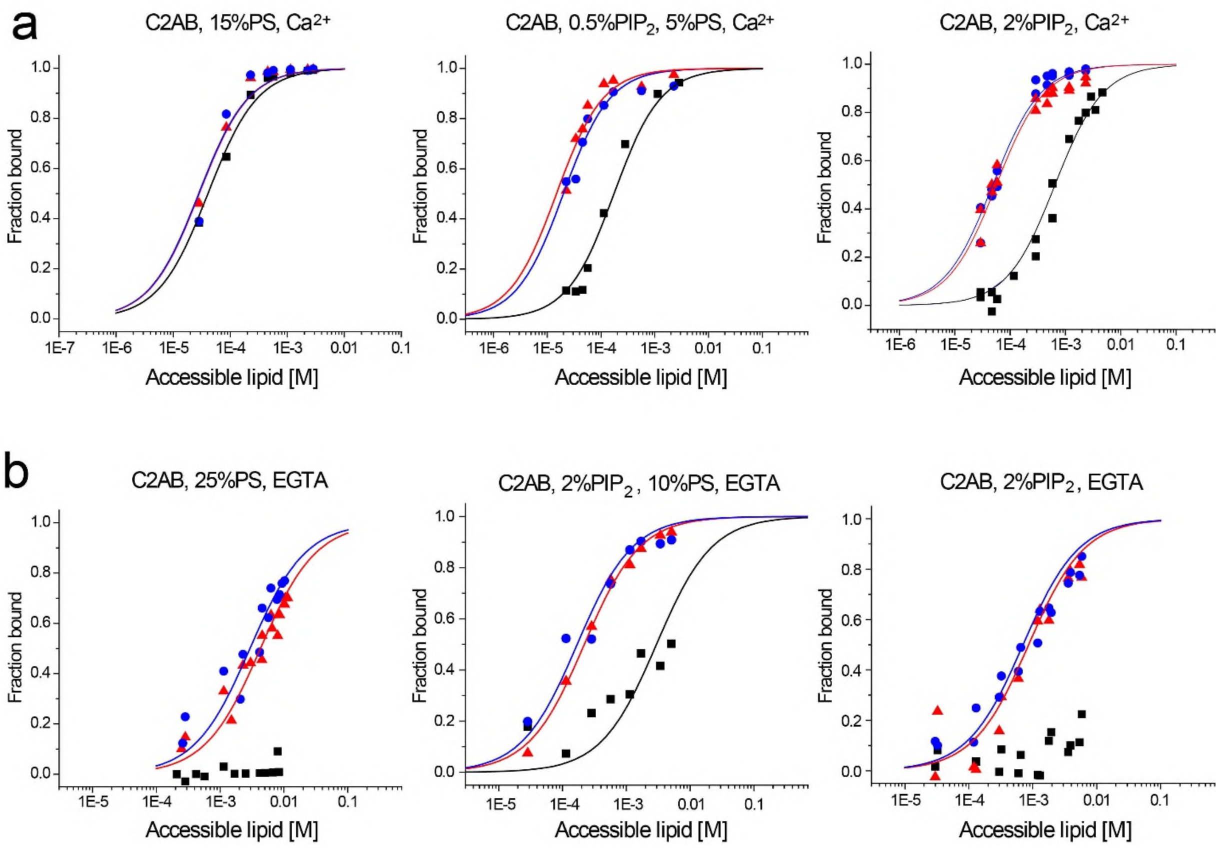

Supplementary Figure 4. *Mutation of the polybasic face but not the arginine apex alters the membrane binding affinity of Syt1C2AB.* Equilibrium membrane binding data were obtained by sedimentation of sucrose loaded vesicles for lipid compositions of PC:PS, PC:PIP_2, and PC:PS:PIP_2 both in the presence (**a**) and absence (**b**) of Ca^{2+} [1]. Both the wild-type (blue circles) and RQRQ mutant (red triangle) have a similar binding affinity in the presence of Ca^{2+} to the 3 lipid mixtures. Unlike the RQRQ mutant, the KAKA mutant in the polybasic face (black squares) exhibits a dramatically weakened membrane affinity, and no binding to either PC:PS or to PC:PIP_2 mixtures was detected in the absence of Ca^{2+} for this mutant.

Supplementary Table 4. *SdFLIC results.* The measured distances for each condition in Ca^{2+} are reported from the mean of n experiments as $d_M \pm$ *standard errors*. Changes from this condition are reported as the mean of the changes from n experiments as *+/-(Δ* $d_M \pm$ *standard errors)*.

C2AB added to	d_M (nm) +/- Δ d_M (nm)	n
**Syx*192/SNAP-25/Syb1-96** in bPC/bPE/bPS/bPIP2/chol (34/30/15/1/20), in +100 μM Ca^{2+}	6.4±0.2	11
+0.4 μM C2AB (WT)	+ (4.7±0.2)	6
+0.4 μM C2AB (R398Q)	+ (4.2±0.7)	10
+0.4 μM C2AB (R398Q/R399Q)	+ (4.1±0.1)	3
+0.4 μM C2AB (K326A/K327A)	+ (3.0±0.2)	7
**Syx*192/SNAP-25/Syb1-96** in bPC/bPE/bPS/chol (35/30/15/20), in +100 μM Ca^{2+}	6.1±0.4	25
+0.4 μM C2AB (WT)	+ (3.7±0.2)	6
**Syx*192/SNAP-25(AAA)/Syb1-96** in bPC/bPE/bPS/bPIP2/chol (34/30/15/1/20), in +100 μM Ca^{2+}	5.6±0.6	14
+0.4 μM C2AB (WT)	+ (2.1±0.5)	7

Supplementary Table 5. *Single SytKD-DCV fusion*. Percent fusion is calculated from the mean of n experiments. Errors are standard errors of repeats. Total number of docking and fusion events are the total numbers from all experiments.

Acceptor SNARE membrane condition	Percent fusion	Total Number of Docking Events	Total Number of Fusion Events	n
Syx/SNAP-25 in bPC/bPE/bPS/bPIP2/chol (34/30/15/1/20), EDTA	23±2	548	127	5
+100 μM Ca^{2+}	24±2	2042	496	5
+100 μM Ca^{2+}/0.4 μM C2ABwt	50±5	1742	841	5
+100 μM Ca^{2+}/0.4 μM C2AB (R398Q)	47±3	388	181	5
+100 μM Ca^{2+}/0.4 μM (R398Q/R399Q)	45±2	440	199	5
+100 μM Ca^{2+}/0.2 μM (K326A/K327A)	35±2	431	153	5

Supplementary Table 6. *Single WT-DCV fusion. Percent fusion is calculated from the mean of n experiments***.** Errors are standard errors of repeats. Total number of docking and fusion events are the total numbers from all experiments.

Acceptor SNARE membrane condition	Percent fusion	Total Number of Docking Events	Total Number of Fusion Events	n
Syx/SNAP-25 in bPC/bPE/bPS/bPI/bPIP2/chol (25/25/15/4/1/30),	41±1	823	340	14
+100 μM Ca^{2+}	64±3	1753	1184	7
Syx/SNAP-25 in bPC/bPE/bPS/bPI/chol (25/25/15/5/30), EDTA	38±3	211	82	5
+100 μM Ca^{2+}	47±3	386	183	6
Syx/SNAP-25 in bPC/bPE/bPS/bPI/bPIP2/chol (32/32/15/1/20), EDTA	26.6±2	516	134	5
+100 μM Ca^{2+}	54.6±2.8	413	220	5
Syx/SNAP-25(AAA) in bPC/bPE/bPS/bPI/bPIP2/chol (32/32/15/1/20), EDTA	16.7±3.2	219	35	4
+100 μM Ca^{2+}	33.6±3.3	185	63	4

Supplementary Table 7. Primer sequences for mutations

Mutant	Primers (from 5’ to 3’)
**Synaptotagmin-1**	
**M173C**	
forward	CCCGCCCTGGACTGTGGGGGTACATCCGATC
reverse	GATCGGATGTACCCCCACAGTCCAGGGCGGG
**T285C**	
forward	CCTCCGCTACGTCCCTTGCGCCGGCAAACTGACTG
reverse	CAGTCAGTTTGCCGGCGCAAGGGACGTAGCGGAGG
**V304C**	
Forward	GAACCTGAAGAAGATGGATTGCGGTGGCTTATCTGATCCC
reverse	GGGATCAGATAAGCCACCGCAATCCATCTTCTTCAGGTTC
**L323C**	
forward	GATGCAGAACGGTAAGAGGTGCAAGAAGAAAAAGACGACG
reverse	CGTCGTCTTTTTCTTCTTGCACCTCTTACCGTTCTGCATC
**T329C**	
forward	AAGAAAAAGACGTGCATTAAGAAGAACACAC
reverse	GTGTGTTCTTCTTAATGCACGTCTTTTTCTT
**F349C**	
forward	GCTTTGAAGTTCCGTGCGAGCAAATCC
reverse	GGATTTGCTCGCACGGAACTTCAAAGC
**E350C**	
forward	GCTTTGAAGTTCCGTTCTGCCAAATCC
reverse	GGATTTGGCAGAACGGAACTTCAAAGC
**R398Q**	
forward	GCCAACCCCCAACGACCCATC
reverse	GATGGGTCGTTGGGGGTTGGC
**R389Q-R399Q**	
forward	CAACCCCCAACAACCCATCGCACAG
reverse	CTGTGCGATGGGTTGTTGGGGGTTG
**K396A**	
forward	CTGAAGAAGGCAAAGACGACG
reverse	CGTCGTCTTTGCCTTCTTCAG
**K326A-K327A**	
forward	CTGAAGAAGGCAGCAACGACG
reverse	CGTCGTTGCTGCCTTCTTCAG
**SNAP25**	
**D51A-E52A**	
forward	GGACTTTGGTTATGTTGGCTGCACAAGGAGAACAACTCG
reverse	CGAGTTGTTCTCCTTGTGCAGCCAACATAACCAAAGTCC

**E55A**	
forward	GGTTATGTTGGCTGCACAAGGAGCACAACTCGATCGTGTCGAAGAAGG
reverse	CCTTCTTCGACACGATCGAGTTGTGCTCCTTGTGCAGCCAACATAACC

Supplementary References

1 Perez-Lara, A. *et al.* PtdInsP2 and PtdSer cooperate to trap synaptotagmin-1 to the plasma membrane in the presence of calcium. *Elife* **5** (2016).
