# Supplementary material for: Conserved arginine residues in synaptotagmin 1 regulate fusion pore expansion through membrane contact
Source: Nat Commun. 2021 Feb 3;12:761. doi: 10.1038/s41467-021-21090-x (PMC7859215; doi:10.1038/s41467-021-21090-x)
Supplement: Supplementary file 3 — Reporting summary [file 41467_2021_21090_MOESM3_ESM.pdf]

nature research

Corresponding author(s): Cafiso, David S.

Last updated by author(s): Dec 22, 2020

Reporting Summary

Nature Research wishes to improve the reproducibility of the work that we publish. This form provides structure for consistency and transparency in reporting. For further information on Nature Research policies, see our Editorial Policies and the Editorial Policy Checklist.

Statistics

For all statistical analyses, confirm that the following items are present in the figure legend, table legend, main text, or Methods section.

n/a	Confirmed	
☐	☒	The exact sample size (*n*) for each experimental group/condition, given as a discrete number and unit of measurement
☐	☒	A statement on whether measurements were taken from distinct samples or whether the same sample was measured repeatedly
☒	☐	The statistical test(s) used AND whether they are one- or two-sided<br>*Only common tests should be described solely by name; describe more complex techniques in the Methods section.*
☒	☐	A description of all covariates tested
☒	☐	A description of any assumptions or corrections, such as tests of normality and adjustment for multiple comparisons
☐	☒	A full description of the statistical parameters including central tendency (e.g. means) or other basic estimates (e.g. regression coefficient) AND variation (e.g. standard deviation) or associated estimates of uncertainty (e.g. confidence intervals)
☒	☐	For null hypothesis testing, the test statistic (e.g. *F*, *t*, *r*) with confidence intervals, effect sizes, degrees of freedom and *P* value noted<br>*Give P values as exact values whenever suitable.*
☒	☐	For Bayesian analysis, information on the choice of priors and Markov chain Monte Carlo settings
☒	☐	For hierarchical and complex designs, identification of the appropriate level for tests and full reporting of outcomes
☒	☐	Estimates of effect sizes (e.g. Cohen's *d*, Pearson's *r*), indicating how they were calculated

Our web collection on statistics for biologists contains articles on many of the points above.

Software and code

Policy information about availability of computer code

Data collection	Fluorescence microscopy setups were controlled by custom built software written in LabView 2016 for Windows (National Instruments). Single vesicle fusion data was analyzed using custom built software written in LabView 2016 for Windows (National Instruments, Austin, TX) Domanska et al THE JOURNAL OF BIOLOGICAL CHEMISTRY VOL. 284, NO. 46, pp. 32158–32166, November 13, 2009. The simulation of the fluorescent release line shapes (Figure 5c) was performed in Matlab_R2019a (Mathworks, Natick, MA). EPR spectra were baseline corrected and normalized using custom Python software provided by David A. Nyenhuis (University of Virginia).
Data analysis	Software to analyze the data was custom built and had been described previously. Single fusion assay: Domanska et al THE JOURNAL OF BIOLOGICAL CHEMISTRY VOL. 284, NO. 46, pp. 32158–32166, November 13, 2009. FLIC intensities were extracted using custom software written in LabView 2016 for Windows (National Instruments, Austin, TX) and the FLIC data fitted using software based on software (FLIC v.0.5) that was kindly provided by Armin Lambacher (Max Planck Institute for Biochemistry, Martinsried/München, D82152 Germany) and described in detail in J. Opt. Soc. Am. B,Vol. 19, No. 6/June 2002. Fits and illustration of fusion data were performed using Igor Pro 8.0 (Wavemetrics). EPR spectra and plots were produced using OriginPro, v.7.5 or v.2021 (OriginLab). Molecular Structures were rendered using PyMOL (Schrodinger)

For manuscripts utilizing custom algorithms or software that are central to the research but not yet described in published literature, software must be made available to editors and reviewers. We strongly encourage code deposition in a community repository (e.g. GitHub). See the Nature Research guidelines for submitting code & software for further information.

Data

Policy information about availability of data

All manuscripts must include a data availability statement. This statement should provide the following information, where applicable:

- Accession codes, unique identifiers, or web links for publicly available datasets
- A list of figures that have associated raw data
- A description of any restrictions on data availability

There are no restrictions on data availability. An Excel Source Data file containing data found in all graphs and EPR spectra (Figures 1-6 and Supplementary Figures 1-4) is available for this manuscript. Any primary data not included in the Source Data file is available from either V.K or D.S.C.

Field-specific reporting

Please select the one below that is the best fit for your research. If you are not sure, read the appropriate sections before making your selection.

☒ Life sciences ☐ Behavioural & social sciences ☐ Ecological, evolutionary & environmental sciences

For a reference copy of the document with all sections, see nature.com/documents/nr-reporting-summary-flat.pdf

Life sciences study design

All studies must disclose on these points even when the disclosure is negative.

Sample size	The sample size for fluorescence and fusion experiments was based upon previous experience.<br>Results from fluorescence experiments are reported as mean from repeated experiments. The number of experiments, mean and standard errors for all experiments are reported in tables as Supplementary Tables.<br>For fusion assays, we also report the total number of recorded events.<br>SDFLIC statistics is explained in detail in Methods Briefly, results are reported as mean +/-std from repeats. Each repeat includes 4-5 acquired images from one sample. On each image ~100 areas were analyzed.<br><br>Progressive power saturation measurements by EPR are well established and have been used to measure membrane position for 30. In cases where the signal-to-noise is good they are highly reproducible. In these cases 2 or 3 measurements on independent samples were taken. In the few cases where the signal-to-noise was poor, additional experimental runs were made.
Data exclusions	No data was excluded.
Replication	The number of independent sdFLIC measurements ranged between 3 and 14. The number of dense-core vesicle fusion experiments ranged between 4 and 14.<br><br>The sample size for EPR measurements (number of independent independent samples and power saturation runs) ranged between 2 and 6.<br>No data was excluded
Randomization	Site specific mutants were designed to examine a testable hypothesis based upon previous studies. The selection of mutants and their allocation into groups was not random but based upon the protein structure and previous functional studies. Randomization of these samples was not carried out and would have been impractical.
Blinding	The students who prepared the samples also made the measurements; as a result, the experiments were not blinded. In the case of the EPR measurements, the student who prepared the samples was the only one qualified to make the measurements. As an educational institution, students do not hand off their samples to staff scientists but are required to learn each step of the research process. In the case of the EPR measurements, there is little the operator can do to change the result. On any single sample, the results from the spectrometer do not vary. Experimental data was analyzed by software and no judgment by the operator is possible. No data was excluded.

Reporting for specific materials, systems and methods

We require information from authors about some types of materials, experimental systems and methods used in many studies. Here, indicate whether each material, system or method listed is relevant to your study. If you are not sure if a list item applies to your research, read the appropriate section before selecting a response.

Materials & experimental systems

n/a	Involved in the study
☒	☐ Antibodies
☐	☒ Eukaryotic cell lines
☒	☐ Palaeontology and archaeology
☒	☐ Animals and other organisms
☒	☐ Human research participants
☒	☐ Clinical data
☒	☐ Dual use research of concern

Methods

n/a	Involved in the study
☒	☐ ChIP-seq
☒	☐ Flow cytometry
☒	☐ MRI-based neuroimaging

Eukaryotic cell lines

Policy information about cell lines

Cell line source(s)	The rat pheochromocytoma PC12 cell line used here was obtained from Edwin Chapman, University of Wisconsin. This cell line is available from American Type Culture Collection, ATCC.org (Manassas, VA)
Authentication	Use of amperometry to detect depolarization-dependent noradrenalin secretion (Liu et al (2005) Mol. Biol. Cell 16: 4463-4472 PMID 9487127); use of cell fractionation to purify large dense core vesicles containing secretogranin II, a known stored secretory product (Liu et al (2002) Mol. Biol. Cell 13: 4266-4278 PMID 12475951); use of electron microscopy to analyze compound exocytosis of large dense core vesicles (Zhang et al (2011) Traffic 12: 600-614 PMID 21272170).
Mycoplasma contamination	Cell lines routine tested for mycoplasma 2002-2017 and were negative for Mycoplasma contamination.
Commonly misidentified lines (See ICLAC register)	No commonly misidentified cell lines were used in this study.
